# Demonstration of mixed properties of RU486 in progesterone receptor (PR)-transfected MDA-MB-231 cells: a model for studying the functions of progesterone analogues

**DOI:** 10.1054/bjoc.2001.2167

**Published:** 2001-12

**Authors:** V C-L Lin, S E Aw, E H Ng, E H-L Ng, M G-K Tan

**Affiliations:** Department of Clinical Research, Department of General Surgery, Singapore General Hospital, 169608, Republic of Singapore

**Keywords:** RU486 morphology cell growth p42/p44 MAPK PR-transfected MDA-MB-231

## Abstract

Progesterone antagonist RU486 (mifepristone) has been implicated for many anti-neoplastic and obstetrical applications. But the compound has demonstrated undesired agonist-like effect depending on cell, tissue and species studied. Using PR-transfected breast cancer cells MDA-MB-231, this report describes the similarities and differences between progesterone- and RU486-mediated effects on cell growth, cell differentiation and, at the molecular level, on the activation of p44/p42 MAP kinases (MAPK). Like progesterone, RU486 inhibited cells growth by arresting the cells in G0/G1 phase of the cell cycle. In contrast to progesterone that induced cell spreading, RU486 induced a multipolar, stellate morphology. RU486-treated cells showed no increase of stress fibers, nor was there any increase of focal adhesions as progesterone-treated cells did. Furthermore, despite of the fact that both compounds inhibited cell growth, RU486 significantly stimulated the activation of p44/p42 MAP kinases whereas progesterone markedly inhibited the activation. Nonetheless, the effects of RU486 were PR-mediated and RU486 was able to antagonize the effect of progesterone on cell growth and focal adhesion. In conclusion, RU486 can act not only as a progesterone antagonist, a progesterone agonist but also induced morphological and molecular changes that were distinct from progesterone-mediated effects in PR-transfected MDA-MB-231 cells. The non-progesterone-like effect of RU486 may be mediated through a pathway that is different from the progesterone-mediated pathway, or it is the result of a blockade of certain critical step(s) in the progesterone-mediated pathway. In any case, undesired side effects of antiprogestin may create clinical complications. PR-transfected MDA-MB-231 breast cancer cells provide a model for studying the functions of progesterone analogues.© 2001 Cancer Research Campaign http://www.bjcancer.com


*British Journal of Cancer* (2001) **85**(12),1978–1986
© 2001 Cancer Research Campaign
doi: 10.1054/ bjoc.2001.2167, available online at http://www.idealibrary.com on IDEAL

http://www.bjcancer.com

### Demonstration of mixed properties of RU486 in progesterone receptor (PR)-transfected MDA-MB-231 cells: a model for studying the functions of progesterone analogues

**VC-L Lin[1], SE Aw[1], EH Ng[2], EH-L Ng[2] and MG-K Tan[1]**

[1]Department of Clinical Research; [2]Department of General Surgery, Singapore General Hospital, Republic of Singapore 169608

**Summary** Progesterone antagonist RU486 (mifepristone) has been implicated for many anti-neoplastic and obstetrical applications. But the compound has demonstrated undesired agonist-like effect depending on cell, tissue and species studied. Using PR-transfected breast cancer cells MDA-MB-231, this report describes the similarities and differences between progesterone- and RU486-mediated effects on cell growth, cell differentiation and, at the molecular level, on the activation of p44/p42 MAP kinases (MAPK). Like progesterone, RU486 inhibited cells growth by arresting the cells in G0/G1 phase of the cell cycle. In contrast to progesterone that induced cell spreading, RU486 induced a multipolar, stellate morphology. RU486-treated cells showed no increase of stress fibers, nor was there any increase of focal adhesions as progesterone-treated cells did. Furthermore, despite of the fact that both compounds inhibited cell growth, RU486 significantly stimulated the activation of p44/p42 MAP kinases whereas progesterone markedly inhibited the activation. Nonetheless, the effects of RU486 were PR-mediated and RU486 was able to antagonize the effect of progesterone on cell growth and focal adhesion. In conclusion, RU486 can act not only as a progesterone antagonist, a progesterone agonist but also induced morphological and molecular changes that were distinct from progesterone-mediated effects in PR-transfected MDA-MB-231 cells. The non-progesterone-like effect of RU486 may be mediated through a pathway that is different from the progesterone-mediated pathway, or it is the result of a blockade of certain critical step(s) in the progesterone-mediated pathway. In any case, undesired side effects of antiprogestin may create clinical complications. PR-transfected MDA-MB-231 breast cancer cells provide a model for studying the functions of progesterone analogues. © 2001 Cancer Research Campaign http://www.bjcancer.com

**Keywords**: RU486 morphology cell growth p42/p44 MAPK PR-transfected MDA-MB-231

There has been increasing interest in the development of antiprogestins for both anti-neoplastic and obstetrical applications (Cadepond et al, 1997; Gravanis et al, 1985; Rocereto et al, 2000). However, some of the progesterone antagonists can have undesired agonist-like effects that are associated with cell, tissue and species specificity. For example, RU486 has been reported to have agonist-like activities in postmenopausal women, as well as in numerous in vitro studies, under conditions in which inhibition would be expected (Gravanis et al, 1985; Meyer et al, 1990; Nordeen et al, 1993). The clinical consequences of the inappropriate agonist-effect of an antagonist can be grave. It is, therefore, of clinical importance to establish appropriate experimental models to identify the agonist-like effects, and to understand the mechanisms by which the effects are mediated.

Antiprogestins exert antagonistic effect by competing with progesterone for PR binding, followed by binding to progesterone response element (PRE). Agonist-PR binding to PRE leads to specific transcriptional responses while antagonist-occupied PR-PRE is non-productive, resulting in the inhibition of agonist activity. However, recent studies reveal more complex mechanisms for antagonist-mediated gene transcription and functional activation. Antiprogestins fall into two mechanistic classes based on their DNA binding properties. Type I antagonist, such as ZK98299, has been shown in vitro to fail to induce PR association with target DNA (Klein-Hitpass et al, 1991; Bocquel et al, 1993; Truss et al, 1994). Type II antagonist, represented by RU486, is capable of inducing high affinity association of PR with target DNA. The DNA binding of RU486 and other type II antagonist is often associated with agonist activities under some circumstances (Meyer et al, 1990; Nordeen et al, 1993; Beck et al, 1996). The presence of cell signaling molecules such as cAMP, enhances these agonist effect (Nordeen et al, 1993; Truss et al, 1994; Sartorius et al, 1994). More recent studies suggest that the recruitment of corepressors or coactivators into the transcription machinery also modulates the relative agonist/antagonist activity of mixed antagonist (Jackson et al, 1997; Simth et al, 1997; Takimoto et al, 1999). For example, coactivator L7/SPA can enhance the partial agonist activity of RU486, whereas the N-CoR and the related corepressor, SMRT, suppress this activity. The net agonist activity seen with mixed antagonists has been shown to be a function of the ratio of coactivators and corepressors.

Studies with PR-transfected MDA-MB-231 cells (Lin et al, 1999) revealed new aspects of RU486-mediated effects. Not only did RU486 antagonize the effects of progesterone on focal adhesion, the compound alone also exhibited agonist activity in that it inhibited cell growth. Furthermore, the compound also induced morphological and molecular changes that were distinct from progesterone-mediated effects. The PR-transfected MDA-MB-231 cells may, therefore, be used as a model system to screen for undesired side effects of antiprogestins that may arise in clinical settings.

*Received 25 September 2000*
*Revised 1 October 2001*
*Accepted 4 October 2001*

*Correspondence to:* VC-L Lin

### MATERIALS AND METHODS

### Chemicals

Progesterone was obtained from Sigma Chemical Co (St. Louis, MO USA). RU486 (mifepristone) and ZK98299 (onapristone) were from Siniwest Holdings, Inc. All tissue culture plastics and reagents were obtained from GIBCO BRL, Life Technologies, Inc.

### Cell culture

MDA-MB-231 cells were obtained from American Tissue Culture Collection (ATCC) in 1995 at passage 28. MDA-MB-231 cells were cloned using 96-well plates by plating 0.5 cell/well. Clone 2 (known as MDA-MB-231-CL2) was selected for transfection studies. All cells were routinely maintained in phenol-red containing Dulbecco's Modified Eagle Medium (DMEM) supplemented with 7.5% fetal calf serum (FCS), 2 mM glutamine and 40 mg/l gentamycin.

### Transfection

MDA-MB-231-CL2 cells were transfected with PR expression vectors hPR1 and hPR2 coding for PR isoform B and isoform A, respectively, in pSG5 plasmid (Kastner et al, 1990). Vector pBK-CMV (Stratagene) containing the neomycin-resistant gene was co-transfected with hPR1 and hPR2. Details of transfection were described previously (Lin et al, 1999).

### Cell growth

Cells ($2 \times 10^4$) in their late exponential growth phase were seeded onto the 6-well plates in phenol red-free DMEM supplemented with 2 mM L-glutamine, 40 mg/l gentamycin and 5% dextran-coated charcoal (DCC)-treated FCS (Test Medium). Treatment of FCS with dextran-coated charcoal was to remove from FCS the endogenous steroid hormones that may complicate the effects of progestins and antiprogestins. Two days later, the medium was replaced with fresh medium containing test compounds that were added from 1000-fold stock in ethanol. This gave a final concentration of ethanol of 0.1%. Treatment controls received 0.1% ethanol only. The medium containing test compounds was changed every other day and the cell numbers were determined by counting on a hemocytometer.

### DNA synthesis

$3 \times 10^3$ cells were seeded onto 96-well plates in Test Medium and were treated with test compounds 2 days later. The effect on DNA synthesis after 48 h treatment were measured by a ELISA kit of bromodeoxyuridine (BrdUrd) incorporation (Boehringer Mannheim, Germany) according to the manufacturer's instructions.

### Cell cycle analysis by flow cytometer

$5 \times 10^4$ cells in 6-well plates were grown in Test Medium for 2 days before they were treated with the test compounds for various time intervals. The cells were then harvested and stained with propidium iodide (PI) in Vindelov's (Vindelov et al, 1983) cocktail (10 mM Tris–HCl, pH 8, 10 mM NaCl, 50 mg PI/l, and 10 mg/l Ribonuclease A, and 0.1% Nonidet P-40) for 20 min in the dark. The stained cells were analyzed in a FACS Calibur flow cytometer (Becton Dickinson) with excitation wavelength of 488 nm. The resulting histograms were analyzed by program MODFIT (Becton Dickinson) for cell distribution in cell cycle phases. The average coefficient of variation (CV) is within 5%.

### Light microscopy

Cells were grown in six-well plates and treated with progesterone and its analogues alone or in combinations in 0.1% ethanol, or 0.1% ethanol for 48 h before they were viewed and photographed under a Zeiss AXIOVERT 35 phase contrast microscope.

### Immunofluorescence microscopy

Cells were grown on glass coverslips in six-well plates and treated with progesterone in 0.1% ethanol or 0.1% ethanol for 48 h. After rinsing with phosphate buffered saline (PBS), the cells were fixed in 4% paraformaldehyde for 10 min and permeabilized with 0.2% Triton X-100 for 10 min. This was followed by incubation with 2% fetal calf serum in PBS for 1 h to block non-specific binding. All the subsequent incubations with Ab were carried out in PBS containing 2% fetal calf serum. For F-actin staining, the fixed and permeabilized cells were incubated with 10 μg/ml FITC-phalloidin in PBS for 1 h at room temperature. For co-staining of F-actin and paxillin, Ab to paxillin were incubated with the cells overnight at 4 ˚C, followed by incubation with FITC-conjugated sheep anti-mouse IgG and FITC-phalloidin at room temperature for 1 h. After washing in PBS, the coverslips were mounted on slides with fluorescence mounting media from DAKO (USA). Stained cells were viewed and photographed using the Zeiss confocal laser scanning microscope model LSM 510.

### Western blotting analysis of p42/44 MAPK

Cells ($1–2 \times 10^6$) grown on 100 mm Petri dishes for 48 h before they were treated with 0.1 μM progesterone or 0.1 μM RU486. Following treatment with RU486 or progesterone, the cells were lysed with 200 μl cold lysis buffer (50 mM Hepes, 150 mM NaCl, 1% Triton X-100, 5 μg/ml pepstatin A, 5 μg/ml leupeptin, 2 μg/ml aprotinin, 1 mM PMSF, 100 mM sodium fluoride and 1 mM sodium vanadate, pH 7.5). After standing on ice for 20 min, the protein supernatants were collected by centrifugation at 13 000 ***g*** for 20 min and the protein concentrations were determined using a protein assay kit (BIO-RAD). 20 μg of the protein were analyzed by Western blotting using ECL kit (Amersham, UK). Following detection of active p42/44 MAPK by antibody against phosphorylated p42/44 MAPK (New England Biolabs), the membrane was stripped in buffer containing 62.5 mM Tris–HCl, pH 6.7, 2% SDS and 100 mM β-mercaptoethanol for 30 min at 55 ˚C. The membrane was re-probed with the anti-p42/p44 MAPKs Ab (New England Biolabs) to determine the amount of total MAPK.

### Statistical analysis

Differences between treatments were tested by Analysis of Variance (ANOVA). Where significant differences were detected by ANOVA, multiple comparisons among means were performed by the least significant difference test. Correlation analysis was performed using the program in Microsoft Excel.

### RESULTS

ER- and PR-negative breast cancer cells MDA-MB-231-CL2 were transfected with PR expression vectors hPR1 and hPR2 as reported

*© 2001 Cancer Research Campaign*

in detail previously (Lin et al, 1999). Eight transfectant clones expressing both PR-A and PR-B were characterized. We have shown that progesterone-mediated effects are similar in these clones. This report concentrates mostly on the studies obtained with clone ABC28 that expressed 660 fmol PR per mg protein.

### RU 486 demonstrated full agonist activity in the inhibition of cell growth of ABC28 cells

Comprehensive studies on the effect of progesterone in the growth of PR transfected MDA-MB-231 cells ABC28 have been previously reported (Lin et al, 1999). We have described that progesterone markedly inhibited the growth of ABC28 cells in a concentration-dependent fashion. As is shown in Figure 1, Type I antiprogestin ZK98299 alone had no effect but was able to reverse 80% of progesterone's inhibitory effect on cell growth. In contrast, type II antiprogestin RU486 alone showed full agonist activity in inhibiting cell growth. Combination of RU486 with progesterone (1 nM) resulted in the highest growth inhibitory effects that can be achieved by either compound alone.

### The effect of RU486 on growth kinetics and cell cycle distribution in ABC28 was similar to that of progesterone

The reductions in cell number of progesterone and RU486 treated cultures were similar following 2, 4 and 6 days of treatment (Figure 2). The treated cultures were reduced to 30% of the vehicle-treated controls after 6 days. Similar to progesterone, RU486 inhibited the growth of ABC28 cells by arresting cells in G0/G1 phase of the cell cycle, with a concurrent reduction in the percentage of S-phase cells (Figure 3). The time course for progesterone and RU486 mediated effects were almost identical. Progressive reduction of S-phase cells

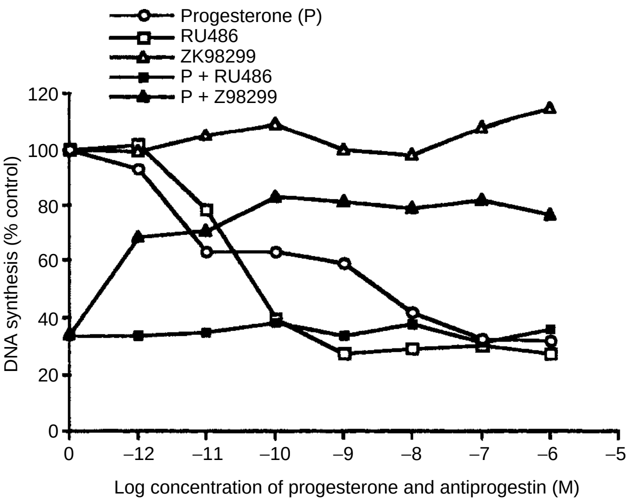

**Figure 1** Effects of antiprogestins RU486 and ZK98299 alone or in combination with progesterone on DNA synthesis in PR-transfectant clone ABC28. The DNA synthesis was determined by ELISA of BrdUrd incorporation. Cells (3000) were plated onto 96-well plates in phenol red-free DMEM supplemented with 5% DCC-FCS. Test compounds-containing medium were added to the cells two days later at various concentrations in 0.1% ethanol. BrdUrd incorporation was measured 48 h later by ELISA according to manufacturer's instructions. Results are the means (*n* = 4) of percentage of vehicle-treated controls. The average standard error of the means is 6.9%. Results are presented for treatment with progesterone, RU486 or ZK98299 alone at various concentrations, or for treatment with 1 nM progesterone (P) in the presence of various concentrations of RU486 or ZK98299

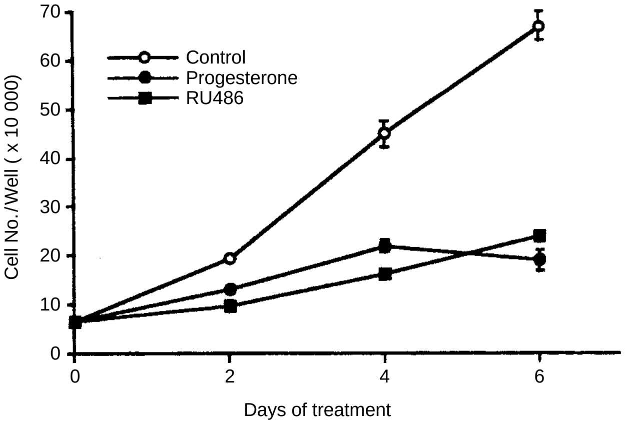

**Figure 2** Effects of RU486 and progesterone on the growth of ABC28 cells were compared over a 6-day period as determined by cell counting. Cells ($2 \times 10^4$) were plated onto 6-well plates in 2 ml of phenol red-free DMEM supplemented with 5% DCC-FCS. 1 nM Progesterone or RU486-containing medium was added to the cells two days later at various concentrations in 0.1% ethanol. The medium containing progesterone or RU486 was changed every two days. The cell numbers were determined by counting on a hemocytometer at 0, 2, 4, and 6 days after treatment. The results are expressed as mean ± SEM, *n* = 4

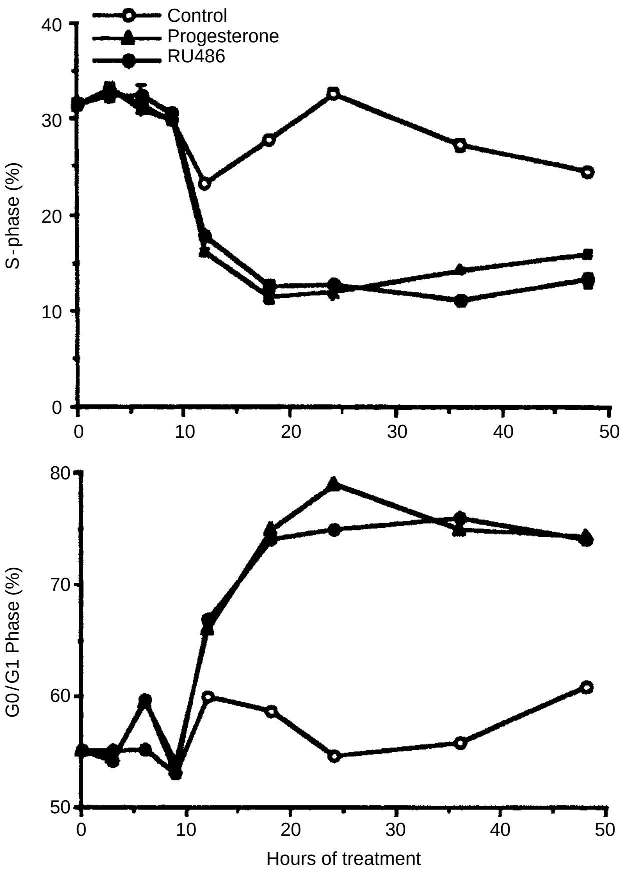

**Figure 3** Effect of RU486 and progesterone on changes in the cell cycle distributions of PR-transfected clone ABC28. $1 \times 10^5$ cells in 6-well plates were grown in 0.5 ml phenol red-free DMEM supplemented with 5% DCC-FCS for 2 days before they were treated with progesterone or RU486 at 1 nM for 24 h. The cells were then harvested and stained with propidium iodide (PI) in Vindelov's cocktail (10 nM Tris–HCl, pH 8, 10 mM NaCl, 50 mg PI/l, and 10 mg/l Ribonuclease A, and 0.1% Nonidet P-40). The stained cells were analyzed in FACS Calibur flow cytometer (Becton Dickinson) with an excitation wavelength of 488 nm. The resulting histograms were analyzed by program MODFIT (Becton Dickinson) for cell distribution in cell cycle phases. The average coefficient of variation (CV) is within 5%. Results are expressed as mean ± SEM, *n* = 4

 *© 2001 Cancer Research Campaign*

began after 12 h of treatment, and this was associated with the concurrent accumulation of cells in the G0/G1 phase. There was approximately 70% reduction of S-phase cells by 24 h. After 48 h of treatment, the effects of both compounds began to diminish possibly due to the depletion of the drugs.

### RU486 elicited distinct morphological changes and had no effect on focal adhesion

Although RU486 showed similar growth inhibitory activity as progesterone in ABC28 cells, the morphological changes induced by RU486 were distinct from those induced by progesterone. As has been reported (Lin et al, 2000) that progesterone-treated cells (Figure 4B) were considerably flattened and more spread than the vehicle-treated controls (Figure 4A). In contrast, RU486-treated cells showed the morphology of multipolar, stellate cells (Figure 4C). Furthermore, progesterone-treated cells showed marked increase in F-actin staining (stress fibers) (Figure 5C) and in focal adhesion protein paxillin (Figure 5D, paxillin staining are regular white spots at the tips of the stress fibers) as compared with vehicle-treated controls (Figure 5A,B). On the other hand, RU486-treated ABC28 cells showed little increase in the staining of stress fibers or focal adhesions (Figure 5E, F).

### RU486 antagonized the effects of progesterone on cell spreading and focal adhesion

As compared with controls (Figure 4A), progesterone antagonist ZK98299 had no effect on cell morphology (Figure 4D) but it inhibited progesterone-mediated cell spreading (Figure 4E). Similarly, RU486 was also a progesterone antagonist in inhibiting the cell spreading induced by progesterone (Figure 4F), and the cells took the morphology of that treated by RU486 alone.

The antagonistic effect of RU486 on cell growth could not be demonstrated in ABC28 cells since progesterone and RU486 are almost of equivalent potency in this cell line. However, RU486 was able to reverse some of the growth inhibitory effect of progesterone in PR-transfectants ABC21 and ABC79 cells in which progesterone was more potent than RU486. As is shown in Figure 6, RU486 inhibited the cell growth of ABC21 and ABC79 cells in a concentration-dependent manner. It also reversed the growth inhibitory effect of progesterone in a concentration-dependent manner. The maximum inhibition is up to the level that was achieved by RU486 alone.

### Progesterone inhibited the activation of p42/44 MAPK whereas RU486 increased the activation p42/p44 MAPK function in a protein kinase cascade that plays critical roles in the cell growth and differentiation. The activation of these proteins is mediated by the phosphorylation of Thr/Tyr residues. In order to understand the differences between progesterone- and RU486-mediated molecular pathways we studied the regulation of activation of p42/p44 MAPK in the treated cells by Western blotting analysis. As is shown in Figure 7 that both phosphorylated p42 and p44 MAPK are detectable but p42 MAPK is the major protein detected in ABC28 cells. p44 MAPK is not visible in the blot probed by anti-p42/44. Interestingly, while both compounds inhibited the growth of ABC28 cells progesterone and RU486 exhibited opposite regulatory roles on the activation of p42/p44 MAPK. Progesterone inhibited the activation of p42/p44 MAPK by as much as 80% of the controls. The inhibition is detectable at 1 h after treatment and the effect persisted for the subsequent 48 h. In contrast, RU486 increased the activation of p42/p44 MAPKs. The effect was notable after 8 h of treatment. The activation continued to increase over time and by 72 h the active p42/p44 MAPKs in RU486-treated cells was about 3-fold of that in the controls. This clearly illustrates that RU486 and progesterone function via different molecular mechanisms in ABC28 cells.

### Effect of RU486 was mediated by PR

The effect of RU486 on cell growth and morphology in ABC28 cells are mediated via PR since RU486 demonstrated no effect on cell growth in PR-negative control transfectant CTC15 (Figure 8). Furthermore, antiprogestin ZK98299 was able to reverse the growth inhibitory effect of RU486 in ABC28 cells (Figure 8) in a concentration-dependent manner. ZK98299 were also able to reverse RU486-induced morphological changes (data not shown). These data substantiate that RU486-induced growth inhibition and morphological changes are mediated by PR.

### DISCUSSION

ABC28 cells is one of the clones established by transfecting PR cDNA into ER-α- and PR-negative breast cancer cells MDA-MB-231 so that the functions of progesterone can be studied independent of estrogens and ER. These cells proved to be an excellent model for delineating the function of progesterone and its analogues. Progesterone was shown to induce remarkable growth inhibition and focal adhesion in the PR-transfected MDA-MB-231 cells ABC28 (Lin et al, 1999, 2000).

RU486 has proved to be a remarkable antiprogesterone and antiglucocorticoid agent in clinical applications since its birth some 20 years ago (Cadepond et al, 1997; Herrmann et al, 1982; Gaillard et al, 1983; Spitz and Chwalisz, 2000). In ABC28 cells, RU486 demonstrated mixed properties. It was shown to be an antagonist in that it inhibited progesterone-mediated cell growth and focal adhesion. The compound was also a progesterone agonist when used alone as it inhibited cell growth and arrested cells in G0/G1 phase of the cell cycle like progesterone did. Furthermore, RU486 showed unprogestin-like effect. It induced the morphology of multipolar, stellate cells whereas progesterone induced cell flattening and spreading in ABC28 cells. In addition, RU486 had no progesterone-like effect on focal adhesion.

More interestingly, the model revealed that RU486 and progesterone exerted their roles differentially at the molecular level in ABC28 cells. p42/p44 MAPKs pathway is generally believed to be the critical route between membrane-bound ras oncogenes and the nucleus to transmit growth and differentiation signals (Hall, 1998; Dent et al, 1998; Sebolt-Leopold, 2000). The activation of these proteins is mediated by the phosphorylation of Thr/Tyr residues. Although both compounds inhibited cell growth, RU486 markedly upregulated the activation of p42/p44 MAPK whereas progesterone inhibited the activation. The results indicate two interesting possibilities. Firstly, since RU486-mediated activation of MAPK is not associated with growth stimulation but growth inhibition, the p42/p44 MAPK pathway may not be a growth signal in MDA-MB-231 cells. Secondly, the downstream of p42/p44 MAPK signaling may be deficient in these cells and that is why it can not signal growth. The results also indicate that RU486- and progesterone-mediated growth inhibitions are mediated by different mechanisms.

RU486-induced morphological changes are also related to distinct molecular events as that of progesterone. RU486-induced

© 2001 Cancer Research Campaign 

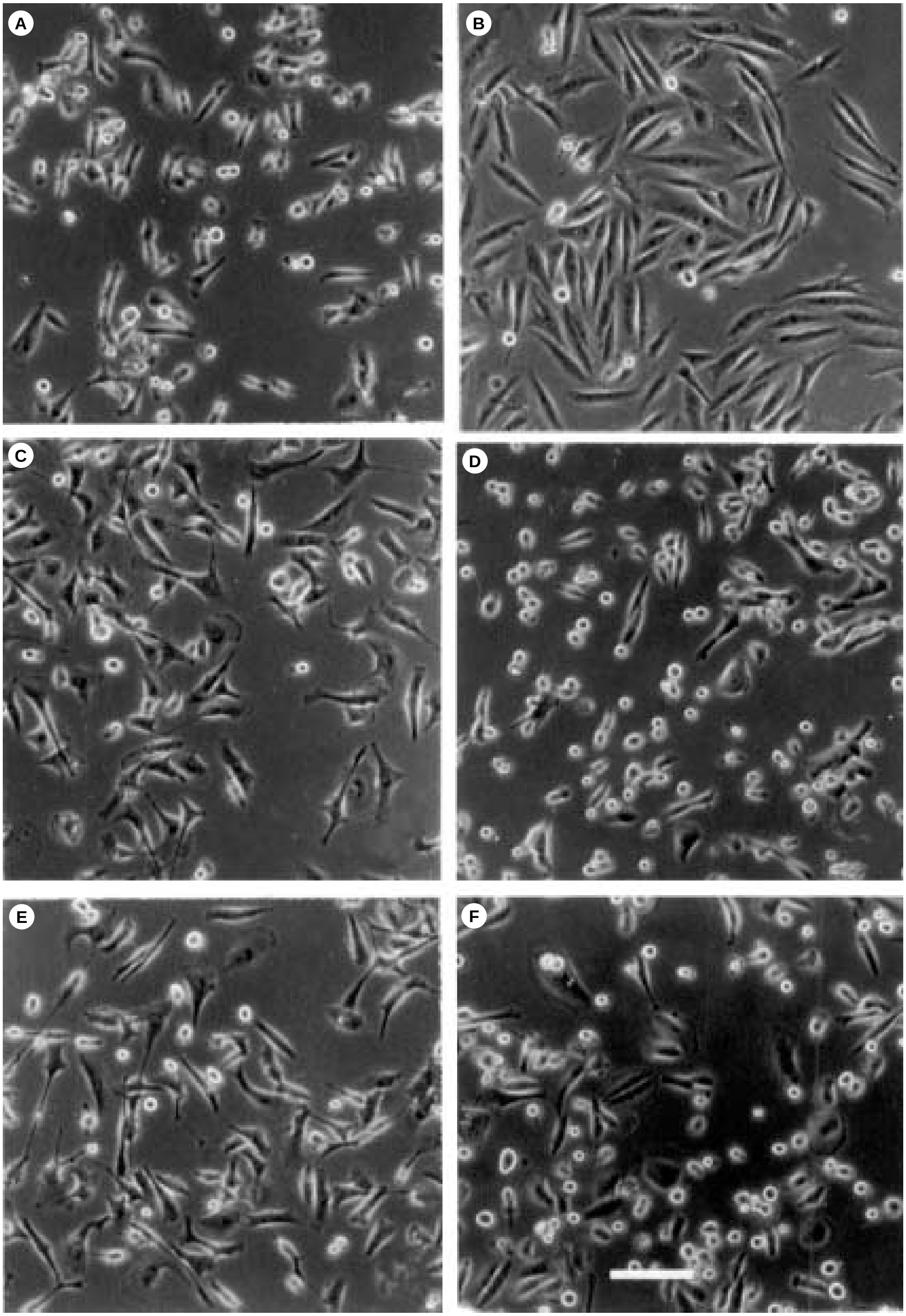

**Figure 4** RU486 induced distinct morphological changes in ABC28 and it was able to reverse progesterone-induced cell spreading. Cells grown in 6-well plates were treated with various compounds at 1 nM in 0.1% ethanol for 48 h before they were viewed and photographed under a Zeiss AXIOVERT 35 phase contrast microscope. Progesterone (1 nM)-treated cells (**B**) are more flattened and spread than the vehicle-treated controls (**A**). RU486 (1 nM)-treated cells took the morphology of multipolar, flattened stellate cells (**C**). RU486 at molar excess inhibited the cell spreading induced by progesterone (**E**). ZK98229 had no effect alone (**D**) but inhibited progesterone-mediated cell spreading (**F**). Bar=100 μM

*© 2001 Cancer Research Campaign*

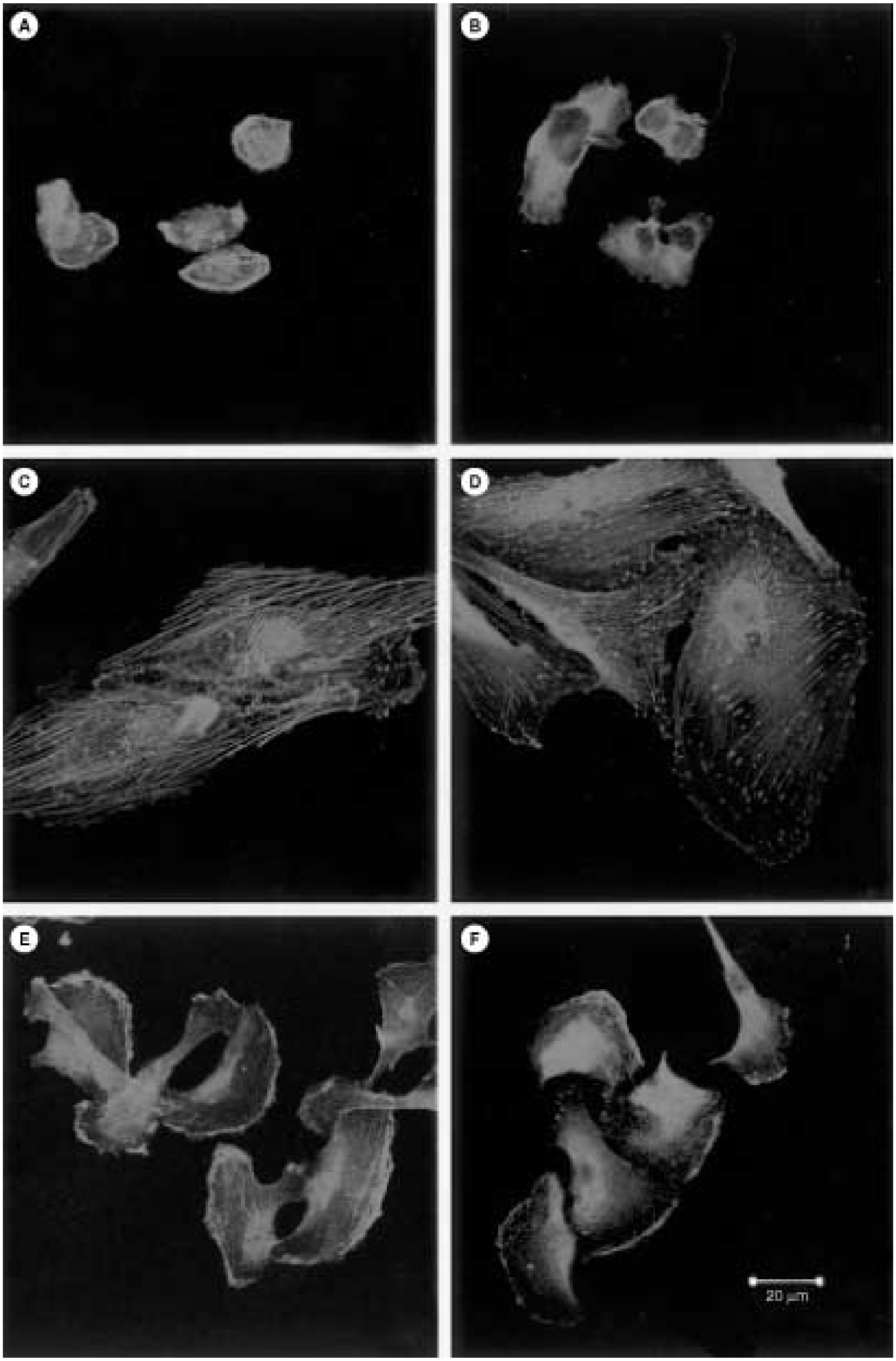

**Figure 5** Effect of progesterone and RU486 on the development of F-actin and formation of focal adhesion in ABC28 cells. ABC28 cells were treated with various compounds at 1 nM for 48 h before they were stained with either FITC-phalloidin (**A**, **C**, **E**), or co-stained with both FITC-phalloidin and focal adhesion protein paxillin mAb which was detected by FITC-conjugated secondary antibody (**B**, **D**, **F**). As compared with vehicle-treated controls (**A**, **B**), progesterone-treated cells showed increased F-actin (**C**) and focal adhesions as identified by focal adhesion protein paxillin (**D**, paxillin staining are regular green spots at the tips of F-actin). RU486-treated cells did not show any increase in F-actin or paxillin staining (**E**, **F**). Bar = 20 μM

*© 2001 Cancer Research Campaign* 

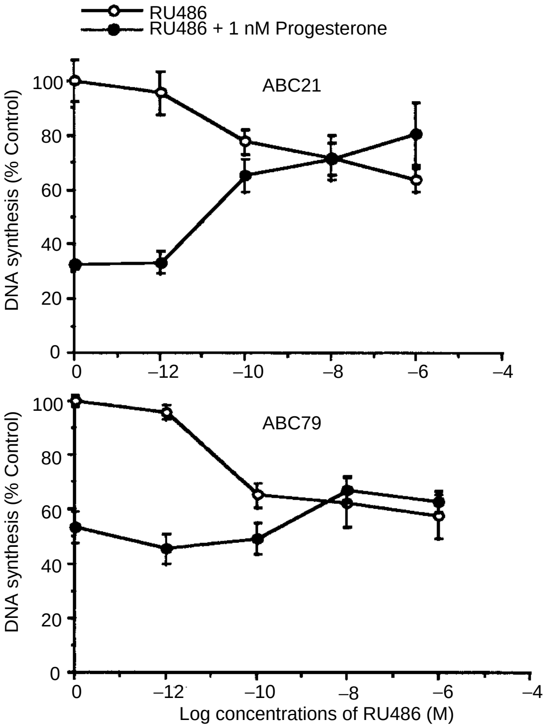

**Figure 6** RU486 was able to reverse progesterone-inhibited cell proliferation in PR-transfected clones ABC21 and ABC79 in which RU486 was less potent than progesterone. RU486 with concentrations range from $10^{-12}$–$10^{-6}$ M was incubated with cells alone, or with $10^{-9}$M progesterone for 48 h before the cells were determined for BrdUrd incorporation using ELISA kit according to manufacturer's instructions. Results are the means (*n* = 4) of percentage of vehicle-treated controls

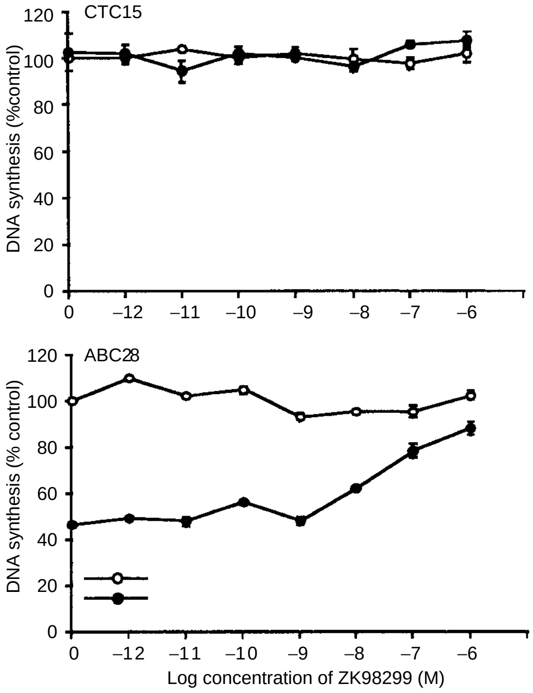

**Figure 8** Antiprogestin ZK98299 was able to reverse the growth inhibitory effect of RU486 in PR-transfectant ABC28 cells and but showed no effect in control transfectant CTC15. Cells (3000) were plated onto 96-well plates in phenol red-free DMEM supplemented with 5% DCC-FCS. ZK98299-containing medium was added to the cells two days later at various concentrations in 0.1% ethanol. BrdUrd incorporation was measured 48 h later by ELISA according to manufacturer's instructions. Results are the means (*n* = 4) of percentage of vehicle-treated controls

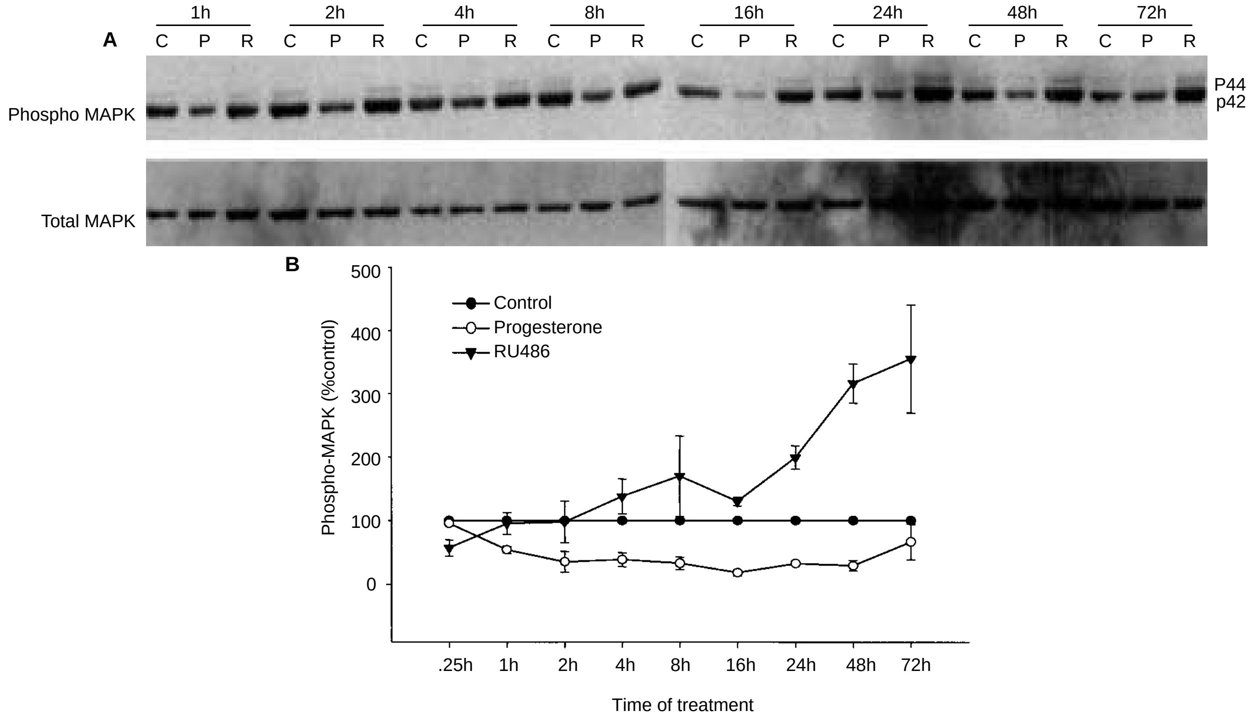

**Figure 7** RU486 enhanced the activation of p42/p44 MAPKs whereas progesterone inhibited the activation in ABC28 cells. ABC28 cells were treated with 0.1 μM RU486, 0.1 μM progesterone or control vehicle (0.1% ethanol) for the indicated periods of time. Whole cell lysate were collected. 20 μg of protein was analysed by Western blotting. Following detection of active p42/44 MAPK by antibody against phosphorylated p42/44 MAPK (ERK 1 and 2), the membrane was stripped and re-probed with anti-p42/p44 MAPKs antibodies to determine the amount of total MAPK. **A** Immunoblot of phosphorylated and total p42/p44 MAPKs. **B** Densitometric data of the immunoblot shown in **A**. The relative amount of activated p42/p44 MAPKs was normalized against the total MAPKs at each time point. The activated p42/p44 MAPKs in the drug-treated groups were expressed as the percentage of vehicle-treated controls

 *© 2001 Cancer Research Campaign*

morphological changes are not associated with increases in the assembly of stress fibres and formation of focal adhesions. In contrast, progesterone-treated cells exhibited a striking increase of stress fibres both in number and diameter, and increased focal contacts as shown by the increase of the staining of paxillin. As was reported in detail previously (Lin et al, 2000) progesterone-induced focal adhesions were associated with distinct increases in tyrosine phosphorylation of focal adhesion protein paxillin and focal adhesion kinase (FAK). The assembly of contractile actin filaments and the associated focal adhesion complexes are generally believed to be activated by a Rho signaling pathways (see review in Hall, 1998).

Several studies have suggested mechanisms by which steroid antagonists inappropriately activate transcription. Cross-talk between antagonist-occupied steroid receptor and cell surface signaling pathways such as the activation of PKA and PKC pathways can switch the antagonist into an agonist (Beck et al, 1996; Sartorius et al, 1994). More recent studies revealed that antagonist-occupied steroid receptors are the targets for the action of endogenous coregulators (Jackson et al, 1997; Simth et al, 1997; Takimoto et al, 1999). The antagonistic effect involves the recruitment of corepressors whereas the binding of coactivators to the receptor-antagonist complexes may result in the exhibition of partial agonist activity. There is also evidence to indicate that RU486-PR complexes may interact with non-PRE sequence. For example, Tung et al (1993) demonstrated using transcription assays that RU486-occupied PR-B was able to activate gene transcription through non-PRE DNA binding sites. Since RU486 is known to be capable of inducing high affinity association of PR with target DNA (El-Ashry et al, 1989; Guiochon-Mantel et al, 1988), it is an interesting possibility that RU486 may also induce a strong association of PR with non-target sequences and subsequently activate ‘unprogestin’-like transcription in ABC28 cells.

It is also interesting to note that the functions of progesterone and RU486 can be differentially mediated by two PR isoforms that arise from the same gene and function to exhibit different transcription regulatory activities in vitro and in vivo (Conneely et al, 2001; Fuhrmann et al, 2000). In the presence of cAMP, RU486 can exhibit inappropriate gene activation in T47D subline expressing PR isoform B but not in cells expressing isoform A (Sartovius et al, 1994). It was proposed that PR-B has a unique third transactivation domain that may confer agonist-like properties in the presence of antiprogestin, whereas PR isoform A exhibits transdorminant negative activity toward PR isoform B (Horwitz et al, 1995).

In summary, the study revealed the mixed properties of RU486 in PR-transfected MDA-MB-231 breast cancer cells and that some of the properties are ‘unprogestin’-like. The signaling pathways mediated by RU486 and progesterone also appeared to be different. It is clinically important to identify these inappropriate side effects of an antagonist. PR-transfected cells ABC28 is an excellent model for studying the functions and for identifying side effects of progesterone receptor modulators as these cells are very sensitive to progestins and antiprogestins.

### ACKNOWLEDGEMENTS

The authors wish to express their sincere thanks to the National Medical Research Council, Republic of Singapore for funding the project, and to Professor Pierre Chambon of Institute of Genetics and Molecular and Cellular Biology, Strasbourg, France for kindly providing the progesterone receptor expression vectors hPR1 and hPR2.

### REFERENCES

Beck CA, Zhang YX, Weigel NL and Edwards DP (1996) Two types of antiprogestins have distinct effects on site-specific phosphorylation of human progesterone receptor. *J Biol Chem* **271**: 1209–1217

Bocquel M-T, Ji J, Ylikomc T, Benhamou B, Vergezac A, Chambon P and Gronemeyer H (1993) Type II antagonists impair the DNA binding of steroid hormone receptors without affecting dimerization. *J Steroid Biochem Mol Biol* **45**: 205–215

Cadepond F, Ulman A and Baulieu E-E (1997) RU486 (Mifepristone): Mechanisms of action and clinical uses. *Annu Rev Med* **48**: 129–156

Conneely OM, Mulac-Jericevic B, Lydon JP and De Mayo FJ (2001) Reproductive functions of the progesterone receptor lessens from knock-out mice. *Mol Cell Endocrinol* **179**: 97–103

Dent P, Jarvis WD, Birrer MJ, Fisher PB, Schmidt-Ullrich RK and Grant S (1998) The roles of signaling by the p42/p44 mitogen-activated protein (MAP) kinase pathway; a potential route to radio-and chemo-sensitization of tumor cells resulting in the induction of apoptosis and loss of clonogenicity. *Leukemia* **12**: 1843–1850

El-Ashry D, Onate SA, Nordeen SK and Edwards DP (1989) Human progesterone receptor complexed with the antagonist RU 486 binds to hormone response elements in a structurally altered form. *Mol Endocrinol* **3**: 1545–1558

Fuhrmann U, Hess-Stumpp H, Cleve A, Neef G, Schwede W, Hoffmann J, Fritzemeier KH and Chwalisz K (2000) Synthesis and biological activity of a novel, highly potent progesterone receptor antagonist. *J Med Chem* **43**: 5010–5016

Gaillard RC and Herrmann W (1983) Clinical use of RU 486: control of the menstrual cycle and effect on the hypophyseal-adrenal axis. *Ann Endocrinol (Paris)* **44**: 345–346

Gravanis A, Schaison, George M et al (1985) Endometrial and pituitary responses to the steroidal antiprogestin RU486 in postmenopausal women. *J Clin Endocr Metab* **60**: 156–163

Guiochon-Mantel A, Loosfelt H, Ragot T, Bailly A, Atger M, Misrahi M, Perricaudet M and Milgrom E (1988) Receptors bound to antiprogestin from abortive complexes with hormone responsive elements. *Nature* **336**: 695–698

Hall A (1998) Rho GTPases and the actin cytoskeleton. *Science* **279**: 509–514

Herrmann W, Wyss R, Riondel A, Philibert D, Teutsch G, Sakiz E and Baulieu EE (1982) The effects of an antiprogesterone steroid in women: interruption of the menstrual cycle and of early pregnancy. *C R Seances Acad Sci III* **294**: 933–938

Horwitz KB, Tung L and Takimoto GS (1995) Novel mechanisms of antiprogestin action. *J Steroid Biochem Molec Biol* **53**: 9–17

Jackson TA, Richer JK, Bain DL, Takimoto GS and Horwitz KB (1997) The partial agonist activity of antagonist-occupied steroid receptors is controlled by a novel hinge domain-binding coactivator L7/Spa and the corepressors N-CoR or SMRT. *Mol Endocrinol* **11**: 693–705

Kastner P, Bocquel M-T, Turcotte B, Garnier J-M, Horwitz KB, Chambon P and Gronemeyer H (1990) Transient expression of human and chicken progesterone receptors does not support alternative translational initiation from a single mRNA as the mechanism generating two receptor isoforms. *J Biol Chem* **265**: 12163–12167

Klein-Hitpass L, Cato ACB, Henderson D and Ryffel G (1991) Two types of antiprogestins identified by their differential action in transcriptionally active extracts from T47D cells. *Nucl Acid Res* **19**: 1227–1234

Lin VC-L, Ng E-H, Aw S-E, Tan MG-K, Ng EH-L, Chan VS-W and Ho GH (1999) Progestins inhibit the growth of MDA-MB-231 cells transfected with progesterone receptor complementary DNA. *Clin Cancer Res* **5**: 395–404

Lin VCL, Ng EH, Aw SE, Tan MG-K, Ng EH-L and Bay BH (2000) Progesterone induces cell spreading and focal adhesion in breast cancer cells MDA-MB-231 transfected with progesterone receptor cDNA. *Mol Endocrinol* **14**: 348–358

Meyer ME, Pmon A, Bocquel M-T, Chambon P and Gronemeyer H (1990) Agonistic and antagonist activities of RU486 on the function of the human progesterone receptor. *EMBO J* **9**: 3923–3932

Nordeen SK, Bona BJ and Moyer ML (1993) Latent agonist activity of the steroid antagonist, RU486, is unmasked in cells treated with activators of protein kinase A. *Mol Endocrinol* **7**: 731–742

Rocereto TF, Saul HM, Aikins JA Jr and Paulson J (2000) Phase II study of Mifepristone (RU486) in refractory ovarian cancer. *Gynecol Oncol* **77**: 429–432

Sartorius CA, Groshong SD, Miller LA, Powell RL, Tung L, Takimoto GS and Horwitz KB (1994) New T47D breast cancer cell lines for the independent study of progesterone B-and A-receptors: only antiprogestin-occupied B-receptors are switched to transcriptional agonists by cAMP. *Cancer Res* **54**: 3868–3877

Sebolt-Leopold JS (2000) Development of anticancer drugs targeting the MAP kinase pathway. *Oncogene* **19**: 6594–6599

Simth CL, Nawaz Z and O'Malley BW (1997) Coactivator and corepressor regulation of the agonist/antagonist activity of the mixed antiestrogen, 4-hydroxytamoxifen. *Mol Endocrinol* **11**: 657–666

Spitz IM and Chwalisz K (2000) Progesterone receptor modulators and progesterone antagonists in women's health. *Steroids* **65**: 807–815

Takimoto GS, Graham JD, Jackson TA, Tung L, Powell RL, Horwitz LD and Horwitz KB (1999) Tamoxifen resistant breast cancer: coregulators determine the direction of transcription by antagonist-occupied steroid receptors. *J Steroid Biochem Mol Biol* **69**: 45–50

Truss M, Bartsch J and Beato M (1994) Antiprogestins prevent progesterone receptor binding to hormone response elements in vivo. *Proc Natl Acad Sci USA* **91**: 11333–11337

Tung L, Mohamed KM, Hoeffler JP, Takimoto GS and Horwitz KB (1993) Antagonist occupied human progesterone B-receptors activate transcription without binding to progesterone response elements, and are dominantly inhibited by A-receptors. *Mol Endocrinol* **7**: 1256–1265

Vindelov LL, Christensen IJ and Nissen NI (1983) A detergent-trypsin method for the preparation of nuclei for flow cytometric DNA analysis. *Cytometry* **3**: 323–327

*© 2001 Cancer Research Campaign*
